# *Dataset on* varietal preferences, gender roles, and rainfall variability in wheat seed systems in Ethiopia

**DOI:** 10.1016/j.dib.2025.112166

**Published:** 2025-10-14

**Authors:** Hom N. Gartaula, Gebrelibanos Gebremariam, Moti Jaleta

**Affiliations:** a International Rice Research Institute (IRRI), Pili Drive, Los Banõs, 4031, Philippines; b Mekelle University, 231, Mekelle, Tigray, Ethiopia; c International Maize and Wheat Improvement Center (CIMMYT), ILRI Sholla Campus, Addis Ababa, Ethiopia

**Keywords:** Ethiopia, Wheat, Gender, Varietal preferences, Seed systems, Climate variability

## Abstract

This dataset provides household- and plot-level information on wheat farmers in Ethiopia, with a focus on gender dynamics, rainfall variability, and farmers’ preferences for wheat varietal traits. The survey was implemented in 2021 by the International Maize and Wheat Improvement Center (CIMMYT) under the Accelerating Genetic Gains in Wheat (AGG) project and covered 1,088 households across the country’s major wheat-growing regions. The questionnaire included modules on household demographics, farm and plot characteristics, wheat production practices, varietal adoption and seed sourcing, gendered decision-making, access to extension services, social capital and networking, food security, dietary diversity, and health. It also documents farmers’ preferences for key varietal traits, including yield potential, drought tolerance, disease resistance, straw yield, baking quality, and nutritional attributes. Household GPS coordinates were used to link survey responses with high-resolution rainfall data, enabling analysis of climate variability and its interaction with household and farm-level outcomes. This dataset provides a valuable resource for researchers and policymakers to examine how gender roles and agroecological conditions shape demand for wheat traits, with implications for designing climate-resilient and gender-responsive seed system interventions in Ethiopia.

Specifications Table

**Table:** 

Subject	Agricultural Sciences
Specific subject area	This article presents the data used for analyzing the wheat trait preferences of men and women farmers in different agroecological regions of Ethiopia.
Type of data	Categorical, string and numeric variables organized in tables, and they are primary data.
Data collection	Individual and household level data were collected using a survey through face-to-face interviews with 984 men and 1,088 women farmers (both women- headed and women in men-headed households) and downloaded CHIRPS rainfall data using GPS Coordinates obtained through the household survey.
Data source location	Data were collected from the three main wheat growing areas (Amhara, Oromia and Southern Nations, Nationalities and Peoples or SNNP) of Ethiopia, and they are stored in the CIMMYT Dataverse.
Data accessibility	Repository name: CIMMYT Dataverse Data identification number: Not applicable Direct URL to data: https://hdl.handle.net/11529/10549168 Instructions for accessing these data: Available in the repository
Related research article	Gartaula, H.N., Gebremariam, G., and Jaleta, M. (2024). Gender, rainfall endowment, and farmers’ heterogeneity in wheat trait preferences in Ethiopia. *Food Policy*, 122: 1-12. https://doi.org/10.1016/j.foodpol.2023.102584

## 1 Value of the Data

This dataset has multifaceted benefits for researchers and policy makers interested in gender-disaggregated data analysis. The data:

• Provides detailed gender-disaggregated data on household roles, decision-making, and preferences for wheat varietal traits in Ethiopia.
• Captures seed acquisition pathways through formal and informal networks, along with social capital shaping varietal adoption.
• Offers insights into gendered access to extension services and institutions, supporting the design of inclusive agricultural programs.
• Includes household food consumption and dietary diversity data, enabling analysis of the nutrition and welfare implications of varietal adoption.
• Contains plot-level information on production practices, input use, and varietal adoption for evaluating productivity and sustainability.
• Links household demographics and welfare indicators to agricultural decisions, gender dynamics, and resilience outcomes.
• Documents farmer demand for wheat traits such as yield, drought tolerance, disease resistance, and nutritional quality, guiding breeding priorities.

## 2 Background

This article presents the dataset underlying the study published in *Food Policy, “Gender, Rainfall Endowment, and Heterogeneity in Farmers’ Wheat Trait Preferences in Ethiopia”* [1]. The study contributes to an emerging body of literature that highlights how intra-household gender dynamics shape agricultural decision-making and influence the adoption of improved technologies [2,3]. In particular, gender differences in preferences for crop traits have been shown to play a crucial role in the development and adoption of new varieties [4,5].

The dataset was generated under the Accelerating Genetic Gains in Wheat (AGG) project, led by CIMMYT in collaboration with Ethiopian national partners. It combines gender-disaggregated household survey data with high-resolution rainfall data to examine how socioeconomic characteristics and climatic variability interact to shape varietal trait preferences. Such integration is important for understanding how gender and environmental endowments jointly affect farmers’ technology adoption decisions, resilience strategies, and welfare outcomes [6,7].

By making this dataset publicly available, we aim to provide a valuable resource for researchers interested in advancing the analysis of gender, climate shocks, and agricultural innovation. The data can be used to test new empirical approaches, extend the findings of Gartaula et al. [1], and generate evidence to inform gender-responsive breeding strategies and agricultural policy design in Ethiopia and beyond.

## 3 Data Description

This dataset includes gender-disaggregated household survey data, available in the file “AGG_HH_Wheat_Survey_2021_Ethiopia.xlsx,” along with rainfall data, provided in “Annual_rainfall_2001_2021_Ethiopia_AGG_Revised.xlsx.” The survey questionnaire used for data collection is also included as “AGG_Wheat_Household_Survey_Ethiopia_2021.pdf” in the CIMMYT data repository (https://hdl.handle.net/11529/10549168 ). Summary of the dataset by category are presented below.

### 3.1 Household data

**Figure 1:**
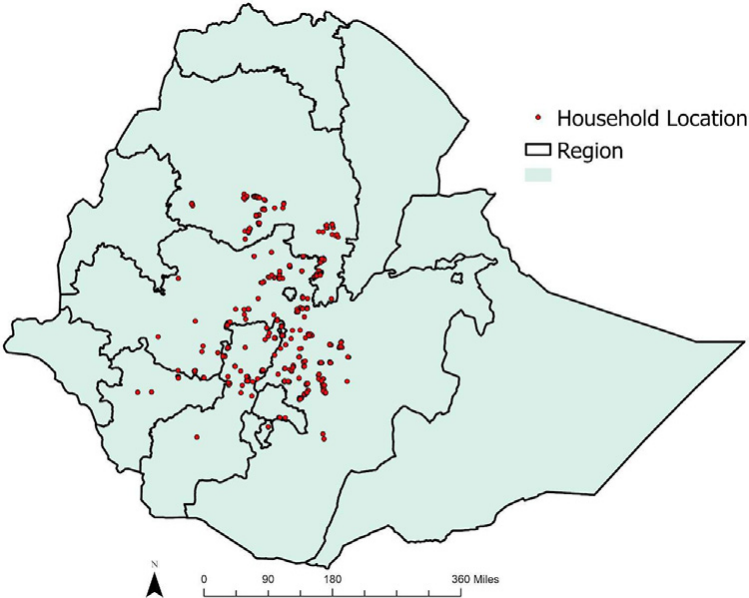
Study area.

Data from 1,088 households located in Ethiopia’s primary wheat production zones were collected during June-August 2021, using a structured questionnaire. The survey was led by the International Maize and Wheat Improvement Center (CIMMYT) in collaboration with national partners in Ethiopia. Using a structured questionnaire, data were collected from randomly sampled households in the three main wheat-growing regions (Amhara, Oromia and Southern Nations, Nationalities and Peoples, or SNNP) of Ethiopia (Fig. 1).

The household-level data provide general household information as well as gender-disaggregated data. The first three sheets contain metadata about the dataset. The sheet named “Questionnaire” includes the first page of the survey questionnaire. The sheet “Variable Description” provides detailed information about all variables in each module, while the sheet “Code Book” lists the variable codes.

The household survey data were collected at both the household level and disaggregated by gender at the individual level. Sheets with names beginning with numerical values indicate variables collected at the household level. Sheets starting with the letter “F” contain data collected from main decision-making female household member in each sample household, whereas sheets starting with “M” contain data from main decision-making male household member. For example, the sheets “F3.1-F3.2” and “M3.1-M3.2” contain information on the module “Socioeconomic and Biophysical Constraints to Wheat Production” for female and male respondents of the same household, respectively. Below we describe the data module by module.

**Module 1: General Household and Farm Characteristics:** This module, comprising sheets “1,” “1.2.8,” “1.2.9,” and “1.3,” provides information on general household and farm characteristics, including household location, composition, and land holdings at the household level.

**Module 2: Plot Information**: This module comprises sheets “2.1a” and “2.1b” and contains information on household-owned plots, including plot size, distance from the household, and the use of inputs and labor on each plot.

**Module 3: Socioeconomic and Biophysical Constraints to Wheat Production:** Both female and male members of the same household making production, consumption, and marketing decisions were interviewed. Data for female **respondents** are in sheets “F3.1_F3.2,” while data for male **respondents** are in “M3.1_M3.2.” This module captures constraints to wheat production, including timely unavailability of improved seeds, high price of improved seeds, poor seed quality, lack of credit access for seed purchase, timely unavailability of fertilizer, high fertilizer cost, lack of credit access for fertilizer purchase, poor quality of chemical fertilizers, timely unavailability of labor, and high wage rates, recorded separately for female and male household members.

**Module 4: Access to New Wheat Varieties**: Both female and male household members making decision on varietal use were interviewed. Data from female respondents are in sheets “F4.1, F4.2, F4.5, F4.6_F4.8, F4.9,” and data from male respondents are in “M4.1, M4.2, M4.5, M4.6_M4.8, M4.9.” This module captures access to improved wheat varieties and varietal preferences, including farmers’ assessments of traits such as yield potential, drought tolerance, disease resistance, straw yield, crop duration, baking quality, nutritional quality, and marketability.

**Module 5: Role in Household Decision-Making Around Production and Income**: This module captures household members’ participation in work activities and their role in decision-making regarding household livelihood activities. Data for female respondents are recorded in sheets “F5” and “F5.1,” while data for male respondents are in “M5” and “M5.1.” For each activity, respondents were asked whether they participated in the activity, who normally makes decisions regarding it, how much influence they had in making decisions, the extent to which they could make their own personal decisions, and how much influence they had over the use of income generated from the activity. The activities covered include overall crop farming (primarily for household consumption), selection of crops, selection of varieties, selection of plots for seed production, seed selection, seed marketing, and allocation of seed for the next season.

**Module 7: Access to Information:** This module captures household members’ access to farming information over the past 12 months, with data collected separately from female and male decision makers in a household. Data from female respondents were recorded in sheet “F7,” and male data in sheet “M7.” Respondents were asked whether anyone in the household accessed information from various sources, including government extension agents, other formal extension agencies, progressive farmers, NGOs, farmer groups and organizations, and traders or dealers. For each source, respondents indicated who made the decision to access the information and, if the information was not accessed, the reason why.

**Module 8: Social Capital and Networking:** The module, which captures household members’ participation in formal and informal institutions related to farming over the past three years. Female respondents’ data are recorded in sheets “F8” and “F8.3,” while male respondents’ data are in “M8” and “M8.3.” The module includes information on whether respondents were members of such institutions, the types of groups they belonged to, the main functions of these groups, the year they joined, their role within the group, whether they are still members, and, if not, the reasons for leaving.

**Module 9: Assets and Amenities:** The data capture household's access to basic amenities and ownership of productive assets over the past 12 months. Female respondents’ data are recorded in sheets “F9.1” and “F9.2,” while male respondents’ data are in “M9.1” and “M9.2.” The module includes information on household amenities such as electricity, piped water, cooking gas, internet, phone connections, private toilets, road access, and housing quality. It also records ownership of productive assets, including houses, plough sets, seed drills, carts, tractors, chaff cutters, sprayers, fishing equipment, water pumps, threshers, and power-tillers, along with details on who made the most recent purchase and in whose name the asset is registered.

**Module 10: Food Insecurity:** The data capture household food insecurity over the past 12 months. Female respondents’ data are recorded in sheet “F10,” and male respondents’ data in sheet “M10.” The module includes information on the number of months household crop and animal production could support consumption and experiences of food insecurity, such as worrying about running out of food, inability to eat nutritious food, limited dietary diversity, skipping meals, eating less than needed, running out of food, going hungry, or going without food for a whole day, including whether these situations worsened due to COVID-19 and associated lockdowns.

**Module 11: Dietary Diversity:** The data capture individual dietary diversity and personal health information for household members. Data from female respondents are recorded in sheets “F11” and data from male respondents are in “M11.” The module includes self-reported health status, COVID-19 infection history, vegetarian status, and consumption of eggs, milk, and milk products. It also records foods eaten in the previous 24 hours across a range of food groups, including cereals, roots and tubers, pulses, nuts and seeds, milk and milk products, flesh foods, eggs, dark green leafy vegetables, vitamin A-rich fruits and vegetables, other fruits and vegetables, oils and fats, condiments and seasonings, savory and fried snacks, sweets, sugar-sweetened beverages, and alcohol.

**Module 12: Food Consumption by the Households** – This module was asked only to main decision-making female household members, with data recorded in sheets “12,” “12.1,” and “12.2.” It captures monthly household consumption over the past 30 days (back from the date of interview) across different food groups, including items that were own-produced, purchased, received as gifts, or obtained in-kind. Respondents were asked to report both the quantity consumed and the equivalent value in ETB.

### 3.2 Rainfall data

We used household-level GPS coordinates to extract monthly rainfall data from the Climate Hazards Group InfraRed Precipitation with Stations (CHIRPS) database for the period 2000–2021. The dataset, provided in the file *“Annual rainfall 2001_2021_Ethiopia_AGG_Revised.xlsx”* (available via the dataverse link), contains annual rainfall totals from 2001 to 2021. These data can be used to construct additional rainfall-based indices for further research. Following the existing literature [[8], [9], [10], [11], [12]], we estimated rainfall shock indicator as stated below.

(1) $$R_{wt}=\left|\frac{r_{wt}-\bar{r_{w}}}{\sigma_{rw}}\right|$$

The rainfall shock (R_wt_) was computed for each household in year t, where r_wt_ represents the observed rainfall during the entire survey season, denotes the household's average seasonal rainfall over the 21-year period, and ​ is the standard deviation of rainfall over the same period.

Households were categorized as experiencing either a rainfall surplus or a rainfall deficit based on the amount of rainfall received during the survey season compared to the historical average. A rainfall surplus was recorded if the rainfall during the survey season exceeded the historical average, while a rainfall deficit was noted if it fell below the historical average.

## 4 Experimental Design, Materials and Methods

### 4.1 Sampling and type of household data

The sampling process followed a three-stage approach:

***Stage 1:*** The initial focus was on regions where wheat production was prominent. Districts with over 2,000 hectares of land under wheat cultivation were identified.
***Stage 2:*** From these identified districts, the dominant wheat agroecologies were determined. Using proportional random sampling, 40 districts were selected across six distinct wheat agroecologies.
***Stage 3:*** Within each sampled district, primary wheat-producing kebeles (the smallest administrative units) were identified. From each kebele, 10 to 18 households were randomly selected from household lists, resulting in a total sample size of 1,088 households.

Interviews were conducted with both a male household member (typically the household head) and a female household member (usually the wife). These participants, referred to as the principal male and principal female household members, provided information on their preferences for wheat traits, along with data on various other variables of interest. Consequently, data were collected from both a man and a woman within the same household.

### 4.2 Survey administration

Skilled enumerators, well-versed in local languages, were tasked with conducting separate interviews with both male and female farmers within households headed by either gender. The objective of these interviews was to document the farmers’ preferences for various wheat traits, alongside capturing information about other crucial factors that pertain to our analysis.

## Limitations

Finally, we acknowledge some limitations in our study. First, we focus on only six wheat traits due to sample size and statistical constraints, though farmers may consider more traits in their decision-making. We suggest that future research explore a broader range of traits in their future research. Furthermore, while rainfall is an important environmental variable affecting wheat preferences and productivity, it is not the only factor at play. Future studies should consider other environmental variables, such as temperature, that may influence wheat trait preferences and productivity (Sun et al., 2024). Such inclusion would provide a more comprehensive understanding of agricultural decision-making both in trait preferences and productivity.

## Ethics Statement

The authors confirm that we have read and followed the ethical requirements for publication in Data in Brief and confirm that the current work does not involve human subjects, animal experiments, or any data collected from the survey households. Before administering the survey, we obtained the Ethics Clearance Certification from the Institutional Research Ethics Committee (IREC) of CIMMYT (Ethics Approval Certificate # IREC.2021.027). To safeguard the privacy of the survey respondents, all collected data were anonymized for analysis. Personal Identifiable Information (PII), such as names and personal details, were replaced with unique codes to ensure confidentiality.

## Credit Author Statement

**Hom N. Gartaula:** Conceptualization, Methodology, Writing – original draft, Writing – review & editing, Project administration, Funding acquisition. **Gebrelibanos Gebremariam:** Conceptualization, Methodology, Software, Validation, Formal analysis, Investigation, Data Curation, Writing – original draft, Writing – review & editing, Visualization. **Moti Jaleta:** Conceptualization, Methodology, Investigation, Writing – original draft, Writing – review & editing, Supervision, Project administration.

## Data Availability

DataverseGender Intentional Wheat Seed Delivery Pathways (Original data).

## References

[1] Gartaula H., Gebremariam G., Jaleta M. (2024). Gender, rainfall endowment, and heterogeneity in farmers’ wheat trait preferences in Ethiopia. Food Policy.

[2] Doss C.R. (2001). Designing agricultural technology for African women farmers: lessons from 25 years of experience. World Develop..

[3] Quisumbing A.R., Meinzen-Dick R., Raney T.L., Croppenstedt A., Behrman J.A., Peterman A. (2014).

[4] Badstue L., Krishna V., Jaleta M., Gartaula H., Erenstein O. (2022). Gender, wheat trait preferences and innovation uptake: lessons from Ethiopia and India. Outlook Agricult..

[5] Jaleta M., Euler M., Gartaula H., Krishna V. (2023). Gender differences in smallholders' socioeconomic networks and acquisition of seed of improved wheat varieties in Ethiopia. Front. Sustain. Food Syst..

[6] Beaman L., Dillon A. (2018). Diffusion of agricultural information within social networks: evidence on gender inequalities from Mali. J. Develop. Econ..

[7] Fisher M., Kandiwa V. (2014). Can agricultural input subsidies reduce the gender gap in modern maize adoption? Evidence from Malawi. Food Policy.

[8] Amare M., Jensen N.D., Shiferaw B., Ciss´e J.D. (2018). Rainfall shocks and agricultural productivity: implication for rural household consumption. Agricult. Syst..

[9] Funk C., Peterson P., Landsfeld M., Pedreros D., Verdin J., Shukla S., Husak G., Rowland J., Harrison L., Hoell A. (2015). The climate hazards infrared precipitation with stations new environmental record for monitoring extremes. Scientific data.

[10] Marenya P.P., Gebremariam G., Jaleta M. (2020). Sustainable intensification among smallholder maize farmers in ethiopia: adoption and impacts under rainfall and unobserved heterogeneity. Food Policy.

[11] Michler J.D., Baylis K., Arends-Kuenning M., Mazvimavi K. (2019). Conservation agriculture and climate resilience. J. Environ. Econ. Manag..

[12] Rocha R., Soares R.R. (2015). Water scarcity and birth outcomes in the Brazilian semiarid. J. Develop. Econ..

